# Colic management: an interview with Professors Staiano and Miele

**DOI:** 10.4155/fsoa-2016-0024

**Published:** 2016-06-06

**Authors:** Annamaria Staiano, Erasmo Miele

**Affiliations:** 1 Department of Translational Medical Science, Section of Pediatrics, University of Naples “Federico II”, Napoli, Italy

**Keywords:** baby's crying, Chamomile, colic, Colimil plus, gastrointestinal disorders, heat-killed, infants, *Lactobacillus acidophilus*, Lemon balm, pediatrician, phytotherapy

## Abstract

**Annamaria Staiano and Erasmo Miele speak to Francesca Lake, Managing Editor:** Following the presentation of the results from an Italian trial on the treatment of infantile colic through the combination of herbal agents and probiotics, Annamaria Staiano (Professor of Paediatrics) and Erasmo Miele (Assistant Professor of Paediatrics; both Naples University, Italy) discuss childhood gastrointestinal disorders. With an introduction to the topic written by Staiano, Miele proceeds to further discuss the topic.

## Professor Staiano

Gastrointestinal disorders are a very common – affecting up to 10% of the pediatric population – and stressful situation not only for children, but also, and especially, for their parents and pediatricians who see a large number of these patients on a daily basis.

Functional gastrointestinal disorders (FGIDs) are divided into early childhood and adolescence disorders.

Early childhood functional disorders are those whose organic cause has not yet been identified. There is an interest from the industry in finding the pathogenic mechanisms underlying these disorders, to provide an effective remedy.

As this problem occurs almost daily, more and more parents are searching for the cause of this disorder. Many of them are linked to morphogenesis and development, and can be easily resolved. However, there is evidence of how these disorders may instead produce remarkable family stress, so as to compromise the quality of life completely. Physicians are thus called upon to prescribe a remedy for FGIDs, even if the condition spontaneously resolves, suggesting therapeutic aids effective and free from effects: thus, what better products are there than a ‘natural’ one, especially for colic disorders?

## Professor Miele

### Q During your speech at the 7th European Pediatric GI Motility Meeting (October 2015 Naples, Italy), you showed an in-press study [1] in which the incidence of colic was linked to disorders such as constipation, vomiting & others. Can we say that there is a cause–consequence relationship? Could there be a shared pathological base for colics & other GI disorders?

There are many longitudinal studies investigating this relationship, since the basis of these functional disorders lies in the same pathophysiological mechanism. As a result, for example, there is a high chance that the children will stop having colic and begin to have gastroesophageal reflux. Then, infants with gastroesophageal reflux in the second year of life may develop specific chronic diarrhea and then irritable bowel.

The most credible mechanism underlying colics is visceral hypersensitivity, or the fact that an infant with colic may have a more sensitive gut that feels the distension of the intestinal lumen with an amount of air that does not lead to anything in a normal child.

The pathophysiological mechanism underlying these disorders is most likely the same, explaining the reason why there often is a shift in the type of FGID affecting the child.

One important message I want to say is that the pain of functional disorder has equal dignity to that due to an organic pathology. The biggest mistake would be to say to parents: “the child has pain and has nothing because it is a functional disorder”. The pain of a functional disorder has the same impact on the quality of life as the pain of an organic disease.

### Q How would you rate colic-related stress for a physician?

For the physician, it is very difficult to manage colic in infants.

Imagine, for example, a young couple with a first child, who cries for more than 3 h a day: the parents are totally desperate. Another example from my experience regards a young physician-father with a daughter often crying and vomiting, who is not able to make a proper diagnosis, despite ultrasound examination to seek the cause of regurgitation and the possible presence of a hypertrophic pyloric stenosis.

This example highlights how difficult it is to manage colic in infants: the father, even though he is a physician, cannot handle the problem.

Doctors find difficulties in effectively managing colic in infants without using imaging techniques and medications that may also have side effects.

Another example: I followed a child who kept crying, with a very anxious mother who arrived at the point of having no more confidence in what I was saying.

The child had nothing but crying and grew beyond the 95th percentile, had some regurgitation with the desperate and anxious mother taking him to accident and emergency several times for checks, eventually changing pediatrician. Then, after a few months, the child ‘healed’ from colic and she contacted me to follow the child again.

One great point of difficulty for us is that if doctors apply the protocols, they very often lose the trust of the parents, who strongly demand a prescription even where there is no need for one. A compromise must then be found, which could lie in the use of ‘natural’ products.

### Q Talking about guidelines & protocols the update of Rome III, in Rome IV, is underway. What are the main recommendations of these guidelines, & how they are to be translated into clinical practice?

The guidelines are useful for diagnosis, but do not give advice about treatment, management and parent reassurance on the benign nature of the gastrointestinal functional disorder.

### Q So, currently there is no treatment guideline? How can a physician translate the Rome criteria into practice?

In clinical practice, applying the Rome III guidelines is rather difficult, since these guidelines assume that the child cries for more than 3 h a day and for more than a week – a condition we do not often see in our practice. On the other hand, the methods used to record children crying are subjective. Thus, the diagnosis of infantile colic in practice is based on the perception that the baby cries a lot, is irritable and has no warning signs of other diseases.

### Q In the absence of a treatment guideline, how do you act in clinical practice?

There is no ‘standard care:’ thus treatment is empirical and depends on the pediatrician.

### Q Going back to the guidelines, where do you see the biggest room for improvement? What would add to Rome III?

The guidelines are primarily on diagnosis. I would add some algorithm and some guidance on the treatment of functional disorders.

### Q There is new & important evidence about using natural approaches in FGIDs: can you summarize your experience, which was translated in the trial you presented at the 7th European Pediatric GI Motility Meeting?

Our experience is definitely positive, because the ‘feeling’ about the product safety is important. I feel confident in recommending a product I think is effective and I feel comfortable prescribing it.

Moreover, the experience of the study and clinical practice has given good results because in colic treatment we see an important placebo effect: this implies the need to choose a product that has no side effects and, in this regard, choosing the combination of tyndallized lactobacillus, chamomile and lemon balm, we can help parents in a safe and confident way, and tell them that we are not prescribing a drug but a herbal product.

### Q Would you please describe briefly your clinical experience & the main results you obtained with the product with the combined approach: herbal extracts & tyndallized lactobacillus, in comparison with others?

We performed a multicenter prospective, randomized, comparative study involving infants with colic, according to Rome III criteria, who were assigned at random to receive a standardized extract of Chamomilla L., Melissa Officinalis L. and tyndallized *L. Acidophilus (H122)* (*ColimilPlus^®^, Milte Italia Spa, Milan, Italy), L. Reuteri DSM 17938* (10^8^ CFU) or simethicone. Treatment was given to subjects for 28 days and they were followed for 4 weeks. A total of 176 patients completed the whole follow-up. The results of our study demonstrated that the median daily crying and fussing time at 28 days was more significantly reduced in the group receiving the standardized extract of Chamomilla L., Melissa Officinalis L. and tyndallized *L. Acidophilus (H122)* when compared with the group receiving *L. Reuteri DSM 17938* (10^8^ CFU) and with the group receiving simethicone. In addition, a significantly higher response to the treatment at 28 days was observed in the first group when compared with the remaining two groups, Therefore, our study suggests that the use of herbal supplements and tyndallized lactobacillus may represent a new therapeutic strategy in the management of colicky infants.
